# *In Vitro* Follicular Activation and Stem Cell Therapy as a Novel Treatment Strategies in Diminished Ovarian Reserve and Primary Ovarian Insufficiency

**DOI:** 10.3389/fendo.2020.617704

**Published:** 2021-02-24

**Authors:** Francesc Fàbregues, Janisse Ferreri, Marta Méndez, Josep María Calafell, Jordi Otero, Ramon Farré

**Affiliations:** ^1^ Institut Clinic of Gynecology, Obstetrics and Neonatology, Hospital Clinic of Barcelona, Barcelona, Spain; ^2^ Institut de Investigacions Biomèdiques August Pi i Sunyer (IDIBAPS), Barcelona, Spain; ^3^ Biophysics and Bioengineering Unit, Faculty of Medicine, University of Barcelona, Barcelona, Spain; ^4^ CIBER de Enfermedades Respiratorias, Madrid, Spain

**Keywords:** in vitro activation, primary ovarian insufficiency, poor ovarian reserve, Hippo signaling, stem cells, poor ovarian response, extracellular matrix, mechanobiology

## Abstract

Usually poor ovarian response (POR) to gonadotropins reflects a diminished ovarian reserve (DOR) that gives place to few recruitable follicles despite aggressive stimulation. The reduction in the quantity and quality of the oocytes with advanced age is physiological. However, some women experience DOR much earlier and become prematurely infertile, producing an accelerated follicular depletion towards primary ovarian insufficiency (POI). Up to now, egg donation has been commonly used to treat their infertility. In the last thirty years, specialists in assisted reproduction have focused their attention on the final stages of folliculogenesis, those that depend on the action of gonadotrophins. Nevertheless, recently novel aspects have been known to act in the initial phases, with activating and inhibiting elements. *In vitro* activation (IVA) combining the *in vitro* stimulation of the ovarian Akt signaling pathway in ovarian cortex fragments with a method named Hippo-signaling disruption. Later, a simplification of the technique designated Drug-Free IVA have shown encouraging results in patients with POI. Another innovative therapeutic option in these patients is the infusion of bone marrow-derived stem cells (BMDSC) in order to supply an adequate ovarian niche to maintain and/or promote follicular rescue in patients with impaired or aged ovarian reserves. In this review, for the first time, both therapeutic options are addressed together in a common clinical setting. The aim of this review is to analyze the physiological aspects on which these innovative techniques are based; the preliminary results obtained up to now; and the possible therapeutic role that they may have in the future with DOR and POI patients.

## Introduction

The ovarian reserve reflects the total of ovarian follicles including non-growing follicles (NGFs) together with those that are growing recruited in the preantral and antral stages phases that can finally reach ovulation. Women are born with a finite pool of ovarian follicles that decreases dramatically during intrauterine life from a peak of about 7 million to 1 million at birth. During childhood the descent continues, so that at the age of menarche about 400,000 persist follicles. Finally at menopause there are only less than 1,000 follicles in the ovaries (1, 2). Moreover, as follicle numbers gradually decline with age, thus a sequence of reproductive events occurs, beginning with reduced fecundity and natural sterility, progressing through menstrual cycle irregularity towards a complete cessation of menstruation at menopause. In theory, this sequence unfolds according to “fixed time intervals” before the subsequent stage (3, 4).

According to these concepts, three different scenarios may occur: a normal decrease of ovarian reserve with age, a lower ovarian reserve set prenatally with an usual postnatal decay, or a decrease of ovarian reserve during adverse postnatal environmental or nutritional challenges (5). Anyhow, the diminished ovarian reserve (DOR) constitutes one of the most important therapeutic challenges in assisted reproduction, since the ovarian response to gonadotropin stimulation is an essential prognostic factor (2, 6, 7).

Primary ovarian insufficiency (POI) that affects 1% of women would be the most extreme clinical manifestation of accelerated ovarian follicular depletion and whose only available reproductive treatment is egg donation (8). Even though menstrual cycles cease in these patients, some of them still contain small residual ovarian follicles.

Recently, due to new physiological knowledge in the earliest folliculogenesis phases, attention has been focused on the possibility of activating dormant follicles in patients with POI.

On the one hand, it has been shown that the balance between activating (Akt stimulatory) and inhibitory pathways (Hippo-signaling) is crucial (9). Moreover it has been demonstrated that the manipulation of these mentioned pathways can have clinical application, such as the disruption of the Hippo-signaling pathway by fragmenting ovarian tissue and activating it by incubating with Akt stimulants in primary ovarian insufficiency patients (POI) (10–12). That technique is named ovarian *in vitro* activation (IVA). Overtime, a modification of the technique has been reported aiming to the disruption of the Hippo-signaling pathway alone and renouncing to the chemical activation of the ovarian tissue (Drug-Free IVA) (13–15).

On the other hand, it has been suggested that infusion of human-derived stem cells could supply a fitting ovarian niche to maintain or promote follicular rescue in patients with impaired or aged ovarian reserves. Human studies propose bone marrow-derived stem cells (BMDSC) both mesenchymal and hematopoietic are feasible candidates to promote ovarian rejuvenation (16, 17).

Follicular waves, quality oocytes and live births were obtained from residual follicles with the IVA approach and BMDSC infusion. Still, more studies are necessary to define the real role of these therapeutic options in patients with DOR and POI.

## Intraovarian Control of Early Folliculogenesis

Ovarian follicular reserve is conditioned by the periodic sequence of the “activation” of primordial follicles that leave their quiescent state. Primordial follicle activation involves recruitment of primordial follicles into folliculogenesis for the eventual selection of one oocyte for ovulation. When this activation is accelerated abnormally the ovarian reserve can be seriously affected (18).

Current strategies for controlled ovarian stimulation focus their effect on growing follicles, whereas dormant primordial follicles cannot be activated by known ovarian stimulation protocols. The knowledge of the biological bases for the awakening of the primordial follicles is essential however, up to this point they are little known. Multiple local factors and intracellular signaling pathways are involved. Activators such as BMP4/7, GDF-9, KIT-ligand, FGF2/7, insulin, GREM1/2, LIF and suppressors factors (AMH, LHX8, PTEN, Tsc1m/TORC1, FOXO3a, YAP/Hippo-signaling, and FOXL2) have been reported to be related to primordial follicle development (9, 19)

Most of the signaling networks and molecules involved in primordial follicle activation have been studied using rodent “lost-on-function” models (20, 21). Currently it is considered that in cases of POI the depletion of ovarian reserve is produced by an exaggerated acceleration of the activation of the pool of primordial follicles.

The maintenance of a correct ovarian reserve will depend on a balance between activating and inhibiting factors. In this sense, recent studies have focused their attention on the phosphatase and tensin homolog (PTEN)/phosphatidylinositol-3-kinase (PI3K)/protein kinase B (AKT)/forkhead box O3 (FOXO3) and Hippo-signaling pathway.

Although the complete mechanism of follicular activation remains undeciphered, studies conducted in mice knockout, have shown that the specific deletion in oocytes of the PTEN and Foxo3 gene promote the activation and the growth of all primordial follicles (22, 23). The PTEN gen encodes a phosphatase enzyme that negatively regulates the PI3K-Akt-Foxo3 signaling cascade (24, 25). It has also managed to promote the activation of primordial follicles sleepers using PTEN inhibitors and/or activators Akt, both in murine and human ovaries. In the ovaries, AKT is a prominent kinase in the PI3K/AKT/mTOR pathway and is expressed in both oocytes and granulosa cells of human follicles (25, 26). AKT has a wide range of substrates with both direct and indirect roles in follicle activation (24, 25).

Coordination of cell proliferation and death is essential for the maintenance of organ size and tissue homeostasis during postnatal life. In mammals, the coordination of both processes is orchestrated by Salvador/Warts/Hippo signal. This signaling pathway consists of different regulators negative effects that act in a cascade of kinases that in ultimately antagonizes the transcriptional coactivator Yes Associated Protein (YAP) and its PDZ-binding motif (TAZ) inducing a suppression of growth (26).

YAP is inactivated by phosphorylation mediated by the Hippo pathway, which excludes it from the nucleus, while the loss of Hippo signaling promotes the accumulation of YAP in the nucleus and an increase in its activity. Once inside the nucleus, the YAP protein acts in coordination with the TAZ transcriptional activators to trigger the expression of CCN growth factors and inhibitors of apoptosis BIRC (baculoviral inhibitors of apoptosis repeat containing). This results in increased proliferation and cell growth. Unlike most activated signaling pathways by extracellular ligands, Hippo is regulated by a network of components related to adhesion, shape and cell polarity (27). These cellular features are mediated by rapid changes in polymerization of the globular actin (form G) to filamentous actin (form F) which induced by fragmenting the tissue and which are the Hippo pathway inhibition triggers. This way is implicated for organ size control, and recent studies provide the theoretical basis to disrupt this pathway that can promote follicle growth.

Studies have shown the deletion of the SAV1 or MST gen in hepatocytes results in augmented livers in mice (28, 29). In cardiomyocytes, deletion of SAV1 leads to enlarged hearts (30) and Hippo-signaling is also engaged in tissue regeneration and expansion of stem cells (31).

Based on initial observations, it has been demonstrated that mechanical signals as ovarian fragmentation, and other forms of ovarian injury led to actin polymerization that disrupt ovarian Hippo-signaling, resulting in nuclear translocation of YAP. Nuclear YAP interacts with transcriptional enhanced associate domain (TEAD) producing transcriptional factors to increase the expression of downstream biochemical signals (cysteine-rich 61, connective tissue growth factor and nephroblastoma overexpressed (CNN) growth factors and baculoviral inhibitors of apoptosis repeat containing (BIRC) apoptosis inhibitors. All this leads to follicle growth (9, 32).

In summary, selective primordial follicles develop to the primary stage under the control of AKT and mTOR signaling (initial recruitment), whereas most primordial follicles remain arrested by dormancy factors. Development of preantral and antral follicle is restrained by the inhibitory Hippo-signaling pathway. Therefore, correct folliculogenesis and maintenance of the ovarian follicular reserve depend on an adequate balance between these two routes.

## Mechanical Signaling in Ovarian Function

Although many hormonal and molecular factors have provided great information about the initial phases of folliculogenesis, the role of the extracellular matrix (ECM) in these processes remains undefined. The role of mechanobiology in the knowledge of ovarian function and the dynamic reciprocity that exists between ovarian cells and their microenvironment has recently gained great interest (33, 34). It is known that while the localization of primordial follicles in the collagen-rich ovarian cortex offers a rigid physical environment that supports follicle architecture and probably plays a role in their survival, ovarian ECM rigidity limits follicular development and therefore oocyte maturation, keeping the primordial follicles in their quiescent stage (35). Besides, it has been shown that growing follicles migrate to the medulla of the ovary, where they encounter a softer, more pliant ECM, allowing its maturation. Thus, changes in the rigidity of the ovarian ECM have a direct effect on follicle development.

The relationship between ovarian physical environment and its functionalism has been demonstrated in some pathological situations, such as primary ovarian insufficiency (POI) and polycystic ovary syndrome (PCOS).

Studies have shown that the distribution of primordial follicles varies depending on the age of the patients. In early ages they would be fundamentally located in the most superficial part of the ovarian cortex. In patients with POI, the few existing primordial follicles would be located very close to the medulla (36). Moreover, in patients with PCOS it has been demonstrated that all pre antral follicles are trapped in cortex which could be explained by an aberrant Hippo-signaling, considered important for adequate follicular growth (37).

Finally, and taking into account all of the above, a recent study in which the remodeling of the mechanical components of the matrisome was investigated has shown that the primordial follicles are located in areas of the ovarian cortex rich in collagen, conferring a rigid physical environment that supports follicle architecture and limits follicle expansion. In addition, the matrisome components vary depending on age and on the different stages of follicular maturation (38).

## Conventional *In Vitro* Activation (IVA) and Drug-Free IVA

Mechanobiology is an emerging field of science based on studying how physical forces and changes in the mechanical properties of cells and tissues are able to regulate their proliferation and differentiation. This scientific discipline attempts to explain how mechanical forces critically regulate cellular biochemistry and gene expression as well as tissue development (39). This effect is established by mechanisms of mechanotransduction. Mechanotransduction, the process by which cells sense and respond to mechanical signals, is mediated by extracellular matrix and cytoskeletal structures. Hippo-signaling pathway is basic in mechanotransduction (40, 41).

Experimental studies in mouse ovary demonstrated that using a PTEN inhibitor and a PI3K activator for two days an increase of FOX3 is produced in oocytes of primordial follicles, suggesting a follicular activation. Subsequent transplantation of these ovaries into ovariectomized hosts revealed the presence of preovulatory follicles and mature oocytes (42). Later studies showed that the fragmentation of the ovarian tissue produced a polymerization of actin and a disruption of the Hippo pathway, favoring follicular growth and the retention of mature oocytes (12, 42)

Considering these experimental studies, conventional IVA was established combining Akt stimulators treatments, ovarian fragmentation and autografting cortical strips by laparoscopic surgery in POI patients (10). After ovarian stimulation, retrieval of mature oocytes, IVF and embryo transfer, a healthy baby was delivered. Conventional IVA was successfully used in POI patients in Japan (11) and China (43).

**Figure 1:**
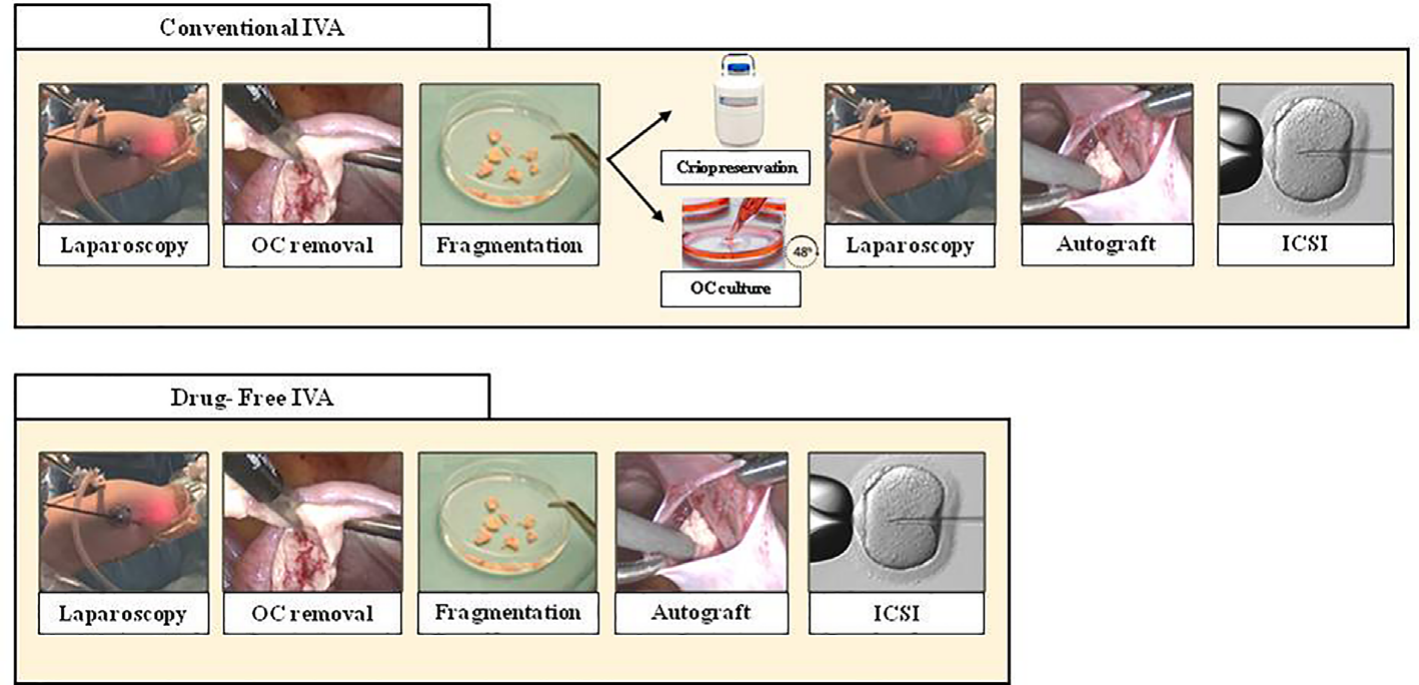
Comparison between Conventional IVA (top line) and Drug Free IVA (bottom line). As seen above, Drug-free IVA avoid second laparoscopy and culture ovarian tissue. OC, ovarian cortex; ICSI, intracytoplasmic sperm injection.

Despite the encouraging results obtained with this technique, it has been hampered by some aspects, mainly the need to perform two laparoscopies and by the possible harmful effect of stimulant substances (44). In this line, a recent modification of the technique has been reported aiming to focus only on the disruption of the Hippo-signaling pathway and avoiding the chemical activation of the ovarian tissue **(**Drug-Free IVA) (**Figure 1**). Fabregues et al. reported a pregnancy for the first time using this technique (13). Another case reported a new pregnancy with this technique (45) and in a large series only a biopsy/scratch of the ovarian cortex was performed, observing follicular waves in 20% of the patients (46). The largest series of patients with POI to whom this technique was applied reported follicle development in 50% of them with oocyte retrieval in five of the 14 patients with four successful pregnancies with a pregnancy rate of 57% per oocyte retrieval and 67% per embryo transfer (14).

Furthermore, Drug-Free IVA has been used preliminarily in POR patients based on the hypothesis that these still have multiple residual follicles that could be activated by mechanical stress. One recent study observed in nine of 11 patients with POR treated with drug-free IVA, multiple growth waves and increases in antral follicle counts were detected after ovarian stimulation treatment. Later retrieval of mature oocytes for IVF allowed 16 embryo transfers in five patients, leading to one live birth, two ongoing pregnancies and one miscarriage. Another patient conceived naturally (15).

In contrast, another recent study has questioned the efficacy of Drug-Free IVA in patients with POR. In 20 patients who were followed up for 10 weeks after the procedure, no significant increase in AFC and AMH levels was observed. Despite this fact, 12 of these patients achieved subsequent pregnancies, which probably reveals a limitation of the study in terms of correct follow-up to assess long-term follicular activity after ovarian cortical fragmentation, followed by autologous grafting under laparoscopic surgery (47). The results achieved so far with IVA and Drug-Free IVA are summarized in **Table 1**.

**Table 1:** Human studies involving IVA and Drug-Free IVA in POI and POR patients.

Author	Ref.	Procedure type	No. of patients	Inclusion criteria	Follicle development/Total	Pregnancies/Total	Live birth: total
Kawamura et al., 2013; Suzuki et al., 2015	(10, 11)	IVA	37	POI	9/37	3/37	2:37
Zhai et al., 2016	(43)	IVA	14	POI	6/14	1/14	1:14
Pellicer et al., 2017**	–	OFFA (Drug-Free IVA)	14	POI	–	3/14	3:14
Zhang et al., 2018	(46)	Biopsy/Scratch	80	POI	11/80	1/80	1:80
Fàbregues et al., 2018; Ferreri et al., 2020	(13, 14)	Drug-Free IVA	14	POI	7/14	4/14	4:14
Mahajan et al., 2019	(45)	Drug-Free IVA	1	POI	1/1	–	–
Kawamura et al., 2020	(15)	Drug-Free IVA	11	POR	9/11*	5/11	2:11 2 ongoing 1 miscarriage

*Patients increased AFC.

**Unofficial data are from conference presentations of stated scientist.

POI, Primary ovarian insufficiency; IVA, in vitro activation; OFFA, Ovarian fragmentation for folicular activation.

## Ovarian Niche Concept

Researchers have studied the structure and functions of the stem cell niche in the hematopoietic system, intestinal system, neuronal cells, spermatogonia and the ovary system (48). The stem cell niche is the micro-environment where the surrounding stem cell survive, and it is composed of niche cells, matrisoma, and cytokines. For many years it has been considered that the aging of the organs is due to the senescence of the stem cells (49, 50). However, some authors indicate that the aging problem is more connected with the aging of the stem cell nests (51, 52). Specifically, in the ovary, ovarian stem cell nest is mainly composed of granulosa cells, vascular endothelial cells,immunologically relevant cells and molecules, surrounding the ovarian reproductive stem cells and regulating their functions. Ovarian germ stem cells belong to the category of adult stem cells and most authors suggest that there are two mechanisms that explain stem cell aging. On the one hand, endogenous aspects that would directly affect them and, on the other, other elements that would be more important that would focus on the exogenous microenvironment. All this is still to be elucidated.

In other words, we know that in increasing age, ovarian function cannot be maintained, despite the ovarian germ stem cell activity and several experimental studies indicated that ovarian function decline is mainly related to the aging of the ovarian germ cell nests but not to the aging of the ovarian germ stem cells (53, 54). All these concepts would serve as a basis to propose the improvement of the niche as a method for the rejuvenation of the ovary.

## Adult Stem Cell Based Therapies to Promote Follicle Development

In recent years, there has been a particular interest in adult stem cell therapy, with the potential to provide the right environment for oocyte development from quiescent primordial follicle. In the human body, the different organs and tissues will present a different proportion of adult stem cells, depending on their turnover. In tissues with high cell turnover, such as the marrow bone or intestine, the population of stem cells is high, and they are usually active throughout life. In this way they are available for repair of these fabrics in great demand. There are other tissues with cell turnover inferior, and where these stem cells remain quiescent and are only activated in the event of injury.

Regarding adult stem cells, research has focused on adult stem cells of hematopoietic origin and of mesenchymal origin. Mesenchymal stem cells (MSCs), relative to tissue cellular, have a very high replication capacity. On the other hand, they have the potential to differentiate themselves when they are cultivated *in vitro* to other cells, such as osteocytes, adipocytes, and chondrocytes (55, 56).

Bone marrow-derived stem cells (BMDSC) are mononuclear cells with low immunogenicity, which makes them ideal for therapy and transplantation. They can renew themselves, to maintain the population of stable stem cells, but they also may have the ability to differentiate from other tissues (adipocytes, cartilage, etc.). These cells can migrate to other damaged tissues and differentiate, after being induced by the release of cytokines from damaged tissues. Yet, they liberate themselves cytokines and growth factors, which promote anti-apoptosis and antifibrosis to restore the ovary, such as VEGF (vascular endothelial growth factor), IGF-1, and HGF (hepatocyte growth factor) (57). Animal studies demonstrated that BMSCs play an important role in restoring injured ovaries in POF induced by chemotherapy in rats (58). Moreover, they also restore ovarian hormone production and reactivate folliculogenesis in a mouse model of POF caused by cisplatin (59, 60). Many researchers have studied whether is possible to regenerate ovarian function. As it has been previously mentioned, adult stem cells have a regenerative capacity through paracrine release of soluble factors: e.g. cytokines, chemokines, growth factors. Thanks to this mechanism, they could take part in the regeneration of dysfunctional ovaries (61, 62). In fact, the soluble factors described in stem cells had already been related to folliculogenesis in ovaries with normal function. Among the growth factors released physiologically by stem cells we find IGF-1, TGF-ß (Transforming growth factor ß), EGF (Epidermal Growth Factor), and FGF1 and FGF2 (Fibroblast Growth Factor1 and 2). FGF-2 has been associated with a positive response to a stem cell therapy, thanks to its action on granulosa cells (63). Given their pluripotency and low immunogenicity, BMSCs are believed to have therapeutic potential for POI.

## Practical Application of BMDSC Therapies

Clinical cases suggested the possible role of stem cells in the recovery of ovarian function. Recovery of fertility and endocrine function of the ovary, after bone marrow transplantation (BMT), in patients with iatrogenic POI after chemotherapy for malignancies has been reported. These cases describe, in addition, spontaneous pregnancies in patients who presented iatrogenic menopause from myeloablative chemotherapy administered in this type of disease.

Hershlag et al. published four cases of induced early menopause after chemotherapy or radiotherapy. All patients received as adjunctive treatment for their disease in addition autologous stem cell transplantation. Two of them with Hodgkin’s Lymphoma and two with infiltrating ductal carcinoma breast, which prior to treatment for their disease conserved regular menses. After treatment, all of them started with amenorrhea and climacteric symptoms, compatible with POI. The authors published spontaneous pregnancies in the 4 patients after having been diagnosed with POI, probably a consequence of autologous transplantation of stem cells (64). Five years later, Veitia et al. reported another case, where a woman regained fertility and achieved spontaneous pregnancy after having been diagnosed with POI of iatrogenic cause. This patient, with Fanconi anemia, received myeloablative chemotherapy and radiotherapy following by an HLA-compatible allogeneic hematopoietic transplant. Years later, it was evidenced a return of menstruation and a spontaneous pregnancy with subsequent birth of a premature (currently healthy) baby. After analyzing the DNA of the baby, that of the mother and that of the donor, it was confirmed the genetic compatibility between the mother and the baby, ruling out the existence of a genetic relationship between the donor and the baby. This makes it quite unlikely that stem cells will produce a replacement direct from the oocytes, if not through the regeneration of the ovarian niche (65).

The first study in which the effect of BMCSD in human ovarian tissue was analyzed as a potential treatment in patients with POI and POR was performed by Herraiz et al. BMCSD were obtained by apheresis after treatment with G-CSF (granulocytic colony stimulating factor) from patients with this diagnosis. These cells were infused in two different groups of immunosuppressed mice, which had been reduced ovarian reserve with different doses of chemotherapy, using more dose to mimic patients with POI, and lower dose to mimic patients with POR. These mice had previously undergone a xenograft human ovarian tissue. In this population of mice, a long-term recovery of fertility was reported, thanks to an increase in ovarian vascularization, proliferation of cells at the level of the ovarian and follicular stroma and a reduction of follicular apoptosis (66).

The preliminary results obtained with the use of stem cells in POI patients seem to depend on two aspects: the stem cells source and the method of administration to reach the ovary. Although adipose-derived stem cells and umbilical cord stem cells have been used in experimental studies, those derived from bone marrow (BMSCs) have aroused greater interest. The main reasons are its low immunogenicity, the ease of being isolated and amplified *in vitro*, and its paracrine and immunomodulatory functions. They migrate to the site of injured tissue and differentiate into specific cell types in the tissue under the induction of certain factors to reconstruct the local micro-environment. By enhancing the function of endogenous cells and regulating the immune response, they are involved in the repair of tissue damage, which makes BMSCs an ideal seed cell for transplantation.

Regarding the administration techniques, human stem cells have been infused into one or both ovaries by various methods, such as transvaginal ultrasound-guided injection (16), ovary injection *via* laparoscopy intra-arterial catheterization of the ovarian artery (67) or a combination of techniques (68, 69). Further studies are needed to determine the most effective approach, although less invasive methods are required for both stem cell collection and instillation.

Gupta et al. described the birth of a live newborn in a 45-year-old postmenopausal woman after applying this technique. They injected the cells derived from bone marrow in the ovary by laparoscopy, infusing them into both ovaries. This way, the patient was exposed to two invasive techniques, in a first place to the puncture of the iliac crest and in a second instead of laparoscopy. After showing follicular growth in both ovaries, they started an ovarian stimulation and with this the pregnancy and subsequent live birth were achieved. They were the first researchers to report a live birth after stem cell therapy in a 45-year-old patient (67). In the same way, Edessy et al. also injected autologous BMSCs opting for the laparoscopic approach. This technique was carried out in 10 patients with POI, with an age between 26 and 33 years, with continued comprehensive checks for one year. The results, likewise, were promising, with the return of menstruation in two patients, one pregnancy, and a healthy live newborn (16).

Gabr et al. used autologous bone marrow mesenchymal cells in 30 women with idiopathic POI, with a range of very broad age between 18 and 40 years. Again, to obtain cells, a medullary aspiration was performed from the iliac crest. In order to introduce them into the ovary, they applied two different techniques in two groups of patients. In the first group, the cells were injected laparoscopically, while in a second group they were injected by catheterization of the ovarian artery (to access one of the two ovaries). After this technique, it was obtained a hormonal improvement in up to 86.7% of the patients, with a durable effect up to two years. Even though 3 patients underwent cycles of IVF, a gestation was achieved, obtained naturally (8).

In a recent and exhaustive study Herraiz et al. evaluated the effect of autologous stem cell ovarian transplant (ASCOT) on ovarian reserve and IVF outcomes of POR patients. The study consisted of BMDSC mobilization into peripheral blood by granulocyte colony stimulating factor (G-CSF) treatment and subsequent collection by apheresis. Cells were injected into the ovarian artery by intra-arterial catheter in one side in such a way that the contralateral ovary was considered as a control. ASCOT resulted in a significant improvement in AFC two weeks after treatment. When optimal ASCOT was considered (increase in AFC of three or more follicles and/or two consecutive increases of AMH levels), ovarian activity improved in 81.3% of women. ASCOT increased the number of stimulable antral follicles and oocytes. However, the embryo euploidy rate was low (16%). Five pregnancies were achieved. Two after embryo transfer and three spontaneously. Interestingly, this study evaluated soluble growth factors after apheresis secreted by circulating BMDSC, and positive effects were associated with the presence of fibroblast growth factor-2 (FGF)-2 and, thrombosponding (THSP)-1. These findings suggest that the treatment with only G-CSF could be effective and to be valid as an alternative therapy, since it is a treatment much less invasive than ASCOT, and therefore with fewer side effects and greater safety for the patient. In the future, the use of these stem cells should be focused on the possibility to release soluble factors and their effect on the ovarian niche (68).

## Conclusion

The study of the factors determining ovarian aging and the pathophysiological mechanisms involved constitutes one of the most interesting challenges of reproductive endocrinology. Knowledge of activating and inhibitory pathways in the early phases of folliculogenesis, as well as the concepts related to stem cell niche in the ovary, represent a new therapeutic scenario in patients with DOR and POI.

Data from animal experimentation has allowed the application of techniques such as IVA and Drug-Free IVA in patients with POI and DOR, reporting several live births. However, in order to confirm these encouraging results randomized studies are needed and therefore should still be considered experimental. The future challenges of these techniques should be focused on predicting which patients would be subject to treatment, since according to the current results only 50% of patients with POI would be able to achieve follicular waves. To know biomechanical aspects of the ovarian tissue of these patients and also to improve aspects of the surgical technique that can make mechanotransduction mechanisms more effective.

Future studies on the physical environment surrounding follicles in diseased ovaries are important to design more refined treatments. In this line, with future adaptation of intravaginal near-infrared cameras and improvement of IVA procedures, it would be possible to select patients with POI and patients with DOR with residual preantral follicles. For a better diagnosis of patients, recent advances in *in vivo* imaging tools could allow monitoring primary to secondary follicles

BMDSCs could be an alternative in ovary regeneration and follicular development in POR or POI patients. ASCOT approach involving the whole BMDSC population seems to be a good approach to treat POR women. This procedure has opened the possibility of proposing stem cell therapy simplified by BMDSCs mobilization into blood by granulocyte colony-stimulating factor (G-CSF) treatment avoiding laparoscopy or intra-arterial catheterization.

These new therapeutic options might soon become a reality.

## Author Contributions

FF conceived, structured, and wrote the text. All authors contributed to the article and approved the submitted version.

## Conflict of Interest

The authors declare that the research was conducted in the absence of any commercial or financial relationships that could be construed as a potential conflict of interest.
